# Gunpowder

**Published:** 1847-06

**Authors:** 


*On Gunpowder.* By Prof. Faraday.—Prof. Faraday briefly noticed the known composition of gunpowder as consisting of —75 parts of nitre, 15 parts of charcoal, 10 parts of sulphur; which, converted into equivalents, give—1 of potassium, 1 of nitrogen, 6 of oxygen, 3-4 of carbon, 0-85 of sulphur, in a state of mechanical mixture. He then proceeded to advert, 1stly, to the *manner of the action of gunpowder.* Gunpowder was described as a solid body, in which a source of enormous power was locked up, capable of being brought into immediate operation whenever wanted: the action thus elicited being itself regulated by human skill with wonderful precision. The respective functions of the sulphur, nitre, and charcoal, in producing the effects of gunpowder, were experimentally shown. The enormous quantity of gas generated by the combustion of gunpowder, irrespective of heat, was exhibited. It was remarked that, on the ignition of gunpowder, though the sulphur begins the combustion, it is not itself burned by the oxygen of the nitre, but unites chiefly with the potassium of that salt to form sulphuret of potassium—a substance which assists in giving to the flame of gunpowder an intense heat. 2ndly. The *amount of heat required for the inflammation of gunpowder* was next adverted to. If gunpowder and steel filings be dropped together through four or five inches of flame, the latter will burn, though the former will not. A flame from the gas was made to play for several seconds on a heap of gunpowder, without lighting it; but, 3rdly, *when actually lighted, gunpowder evolves very great heat.* It is to the immense heat produced on the solid products of the combustion of gunpowder, that the certainty of its complete combustion is greatly owing. In this respect gunpowder differs characteristically from gun-cotton. The latter fires at a heat which would not affect the former; but produces, by its combustion, a degree and condition of heat much less communicable to other bodies. 4thly. The *effect of the heat generated, independent of the chemical change from the solid to the gaseous or vaporous state*, namely, that due to mere expansion or increased elasticity producing the effects of gunpowder, was adverted to. This was illustrated by the violence with which a mixture of one volume of oxygen with two of hydrogen gas bursts the vessel which contains it, solely in consequence of the heat elicited during their combination. This is manifest from the fact that the space occupied by the uncombined gases, is greater by one-half than that taken by the resulting steam. 5thly. Prof. Faraday laid great stress on the *effect of the granulation of gunpowder.* To this condition of gunpowder, presenting, as it does, a number of separated surfaces of size just sufficient to become surrounded with flame at the same instant of ignition, much of the disruptive or projectile effect of gunpowder was ascribed. It was shown that, without that porosity which its division into grains imparts to a mass of gunpowder, the explosion of the whole could not be instant nor simultaneous. This was proved by bringing a piece of mill-cake successively into the condition of grain powder and of meal powder. The slow combustion of the solid meal powder fuse was compared with the quicker inflammation of the hollow rocket, and the instant inflammation of the charge of a gun. All these effects are related to the condition of the interior of the gunpowder in respect to its permeability by the flame of the first particles ignited. Then, as to its exterior condition, it was shown that the tardy burning of the miner's fuse is due to the granular state of the powder in its case being counteracted by the pressure of the strands of rope wrapped very tightly round it; while, on the other hand, in the cracker of the firework-maker, a similar train is instantly fired throughout, because it has a loose jacket all over it, and, in the burning of the common cracker, an alternation of these effects is produced. In conclusion, Prof. Faraday dwelt on the great importance of *time* in producing the effects of gunpowder. Contrasting the action of gunpowder with that of fulminating mercury and silver, or of those still more fearfully explosive compounds, the chlorides and iodides of nitrogen, Prof. Faraday showed that, if the explosion of gunpowder were really instantaneous, it would be useless for all its present applications. As it is, however, whenever gunpowder is fired in the chamber of a gun, it does not arrive at the full intensity of its action until the space it occupies has been enlarged by that through which the ball has been propelled during the first moment of ignition. Its expansive force is thus brought down and kept below that which the breech of the gun can bear, whilst an accumulating, safe, and efficient momentum is communicated to the ball, producing the precise effects of gunnery. This manageable action was contrasted with the effect of a morsel of iodide of nitrogen put on a plate, and exploded by being touched by the extremity of a long stick. The parts immediately in contact with the iodide were shattered,—*i. e.*, the end of the stick was shivered, and the spot in the plate covered by that substance, was drilled as if a bullet were fired through it, yet no tendency to lift the stick was felt by the hand,—whereas the comparatively gradual action of gunpowder lifts and projects those weaker substances, wadding and shot, which give way before it.—*Trans. Royal Inst.—Athen.—Jour. Franklin Inst.*

